# A Review of Cranial Remolding Orthosis Treatment for Babies with Combinational Deformational Plagiocephaly with Brachycephaly and Development of a New Classification Scale

**DOI:** 10.3390/children13050625

**Published:** 2026-04-30

**Authors:** Jill L. Findley, Anna L. Trebilcock, Jeffrey A. Kasparek, Melody M. Gordon, John T. Reets, Stephen P. Beals, Timothy R. Littlefield

**Affiliations:** 1 Cranial Technologies, Inc., Tempe, AZ 85284, USA; 2 Center for Cleft and Craniofacial Care, Phoenix Children’s Hospital, Phoenix, AZ 85013, USA

**Keywords:** plagiocephaly, brachycephaly, cranial remolding orthosis, cranial deformity, helmet therapy, treatment outcomes

## Abstract

**Background/objectives:** This comprehensive study of infants with combinational deformational plagiocephaly with brachycephaly (cDPB) and combinational deformational brachycephaly with plagiocephaly (cDBP) examined the effectiveness of a custom, thermoplastic vacuum-formed cranial remolding orthosis and identified clinical predictive factors that affect treatment outcomes. In addition, a standardized classification scale for babies with combinational head shapes was developed for this study. **Methods:** This was a retrospective study of patients who were treated by Cranial Technologies between January 2014 and March 2025 and met the following inclusion criteria: (a) presented with a Cranial Vault Asymmetry Index (CVAI(S)) > 3.5 AND Cephalic Index (CI) > 90, (b) were compliant with the treatment protocol, (c) were nonsyndromic, and (d) did not have craniosynostosis. Infants with isolated plagiocephaly or isolated brachycephaly were excluded. Multiple linear regression (MLR) analyses and paired t-tests were used to model the effects of clinical predictive factors on CVAI(S) and CI outcomes and determine the change between pre- and post-treatment cranial anthropometric measurements, respectively. **Results:** N = 82,326 infants met the inclusion criteria for this study. The mean overall reduction in CVAI(S) was −3.64 (*p* < 0.001) and for specific age groups it was as follows: 3–4 months −5.274 (*p* < 0.001), 4–6 months −4.074 (*p* < 0.001), 6–8 months −3.262 (*p* < 0.001), 8–11 months −2.692 (*p* < 0.001), and >11 months −2.247 (*p* < 0.001). The mean overall reduction for CI was −3.84 (*p* < 0.001) and was not related to age. In terms of clinician-rated outcomes, 95–100% of babies who entered treatment at less than 6 months of age had a “good” or “great” outcome, while the “good” or “great” success rate dropped to less than 19% for babies who started treatment after 11 months of age. MLR (Adj. R^2^: 0.582) identified the following factors as significant predictors (*p* < 0.001) for change in CVAI(S): entry age (β = 0.008), left-sided plagiocephaly (β = −0.420), initial cephalic index (β = −0.056), initial CVAI(S) (β = −0.479), prematurity (β = −0.189), and the presence of torticollis or neck muscle involvement (β = 0.053). A second MLR for change in CI (Adj. R^2^: 0.217) observed significance (*p* < 0.001) for the following predictors: initial CI (β = −0.335), left-sided plagiocephaly (β = −0.332), multiple birth (β = −0.290), male sex (β = −0.158), initial CVAI(S) (β = −0.049), premature (β = −0.086), and neck muscle involvement (β = −0.106). **Conclusions:** CRO treatment for cDPB/cDBP resulted in highly significant improvement in both CVAI(S) and CI across all age groups with the youngest babies requiring the shortest treatment durations and demonstrating more favorable results, especially in correcting asymmetry. Overall, the mean reduction in CVAI(S) varied significantly between age groups, with younger babies experiencing greater change in CVAI(S) scores, whereas CI did not demonstrate the same relationship with entry age. This study proposed new terminology for infants who present with elements of both plagiocephaly and brachycephaly. The use of standardized terms such as cDPB/cDBP to describe a combinational head shape will enhance comparability across future studies, thus enabling better outcome reporting.

## 1. Introduction

The core terminology used to define nonsynostotic positional head deformities includes deformational plagiocephaly, brachycephaly, and scaphocephaly (dolichocephaly). The term deformational plagiocephaly describes a head shape characterized by occipital flattening with associated anterior displacement of the ear and prominence/bossing of the ipsilateral forehead, resulting in a parallelogram-shaped skull when viewed from above [1,2,3]. Deformational brachycephaly describes a head shape with symmetrical occipital flattening, a reduced anterior–posterior cranial dimension, and increased biparietal width, producing a broad and shortened skull shape [1,2]. Deformational scaphocephaly refers to a head shape characterized by an elongated and narrow skull, with increased anterior–posterior length and decreased biparietal width [2].

The nomenclature used to describe infants with nonsynostotic skull deformities with a combination of both asymmetry and disproportionality varies widely in the literature, highlighting the need for standardized terminology. For example, the names used to describe this condition include “asymmetrical brachycephaly;” [4,5,6] “combined plagiocephaly and brachycephaly;” [7] “plagiocephaly brachycephaly;” [8] “brachycephaly plagiocephaly;” [8] “deformational plagiocephaly and brachycephaly;” [9] “mixed form;” [10] “posterior plagiocephaly and brachycephaly;” [11] and “deformational asymmetrical brachycephaly.” [6]. For this study, we use and propose the new term “combinational deformational plagiocephaly with brachycephaly” (cDPB) to describe a combinational head shape where plagiocephaly is the more predominant deformity (as determined by anthropometric measures) and “combinational deformational brachycephaly with plagiocephaly” (cDBP) to describe a combinational head shape where brachycephaly is the more predominant deformity.

There is a lack of clinical consensus on a severity classification system for categorizing babies with cDPB/cDBP. The need for the development of a classification system for differentiation of deformational plagiocephaly, deformational brachycephaly, and a combinational head shape has been recognized [4,8,12]. Hinken et al. suggested a classification system using CVA and CI to differentiate among plagiocephaly, brachycephaly, and a combination of both, where plagiocephaly is defined as CI < 90% and CVA > 1 cm; brachycephaly is CI > 90% and CVA < 1 cm; combination of plagiocephaly and brachycephaly is defined by a CI > 90% and a CVA > 1 cm; and a borderline head shape where CI is 90% or less and CVA < 1 cm [12].

Similarly, Pinyot et al. developed a new classification scale that uses CVA and CI parameters to classify combinational head shapes, where head shape types were divided into 14 different groups [8]. This scale proposes that the predominant deformity, based on the greatest anthropometric value, is listed first in the naming convention, a nomenclature we follow in our recommendations. Graham et al. tabulated subject counts with different head shape types using CVAI(L) and CI, using the Children’s Healthcare of Atlanta Plagiocephaly Severity Scale (CHOA Scale) [4]. CVAI(L) is listed as the row variable to describe asymmetry severity, and the CI as the column variable to describe the severity of disproportionality. Their CI scale was as follows: CI < 90% as normal, 90% ≤ CI ≤ 93% as mild, 93% < CI ≤ 97% as moderate, and CI > 97% as severe. However, they acknowledged that there is no universally adopted scale for CI or brachycephaly, and that normative values vary in the literature. Moreover, normocephalic values and diagnostic thresholds for brachycephaly may differ across cultures and ethnicities; for example, Asian infants often demonstrate higher CI values.

There is growing evidence in support of the clinical efficacy of CROs in treating both deformational plagiocephaly and/or brachycephaly, including infants with a combination of both [13,14,15,16,17,18,19,20,21,22,23,24,25]. A retrospective chart review of 275 babies (n = 118 babies with cDPB/cDBP) who were treated with a CRO revealed that the babies with cDPB/cDBP (combinational head shapes with aspects of both plagiocephaly and brachycephaly) made progress in asymmetry correction which were similar to the rate of change in the isolated plagiocephaly group (n = 107) and that the cephalic index changed more rapidly in the isolated brachycephaly group (n = 32) than the cDPB/cDBP group [26].

Several studies have identified clinical predictive factors associated with plagiocephaly and/or brachycephaly outcomes [9,19,27,28]. A study of 141,513 babies, inclusive of all head shape types, identified that older age, greater severity at initiation of treatment, and noncompliance with the helmet treatment wearing schedule were each associated with worse craniometric and provider-reported outcomes [9]. A large retrospective study of 27,990 babies with isolated plagiocephaly found that the following predictors were associated with greater reduction in CVAI(S) scores: (1) younger entry age (*p* < 0.001, β = 0.01), (2) larger initial CVAI(S) scores (*p* < 0.001, β = −0.43), (3) left plagiocephaly (*p* < 0.001, β = −0.36), and (4) and the absence of torticollis (*p* < 0.001, β = −0.17) [27].

The purpose of this research is to evaluate the efficacy of CRO treatment specifically for improving asymmetry and disproportionality in infants with combinational head shape deformities (cDPB/cDBP), identify factors that influence treatment outcomes for this specific type of head shape deformity, and refine a standardized severity classification system/terminology for cDPB/cDBP.

## 2. Materials and Methods

### 2.1. Study Design

This study was a retrospective study of infants with combinational deformational plagiocephaly with brachycephaly who completed treatment with a CRO (DOC Band^®^, Cranial Technologies, Inc., Tempe, AZ, USA) at any clinic throughout the United States from January 2014 to March 2025. This CRO is a custom-made device designed to direct growth by using gentle holding pressures on bossed bone areas of the skull and allow for redirection of growth in areas of flattening. It comprises an interior layer of closed-cell polyethylene foam and an exterior thermoplastic copolymer shell (polypropylene-polyethylene blend). Specialized clinicians oversee fitting and adjusting the orthosis, as well as monitoring growth and correction to ensure optimal outcomes. Growth adjustments are made to the CRO every 1 to 3 weeks, on average, depending upon the infant’s age, with younger babies requiring more frequent growth adjustments due to their more rapid skull growth velocity.

### 2.2. Data Source

Patient data were queried from Cranial Technologies’ internal electronic health record (EHR) system (Intergy—Greenway Health, Tampa, FL, USA) and its 3-dimensional Digital Surface Imaging^®^ (DSi^®^) program using Microsoft SQL Server Management Studio (SSMS, Redmond, WA, USA). The DSi^®^ is a 3D imaging system designed specifically for infants with cranial deformation, which generates 3D rendered images of the infant’s head shape. These 3D head shapes are processed through a proprietary program (Landmarks3D^TM^ Rev 06 Tempe, AZ 85284), which analytically identifies all key cranial landmarks which are then used to calculate anthropometric measures. Collection and analysis of the retrospective data did not involve direct contact with patients, their families, or their physicians. Institutional Review Board (IRB) approval was obtained from Advarra IRB in April 2025 with a waiver of informed consent.

### 2.3. Subject Identification

**Figure 1:**
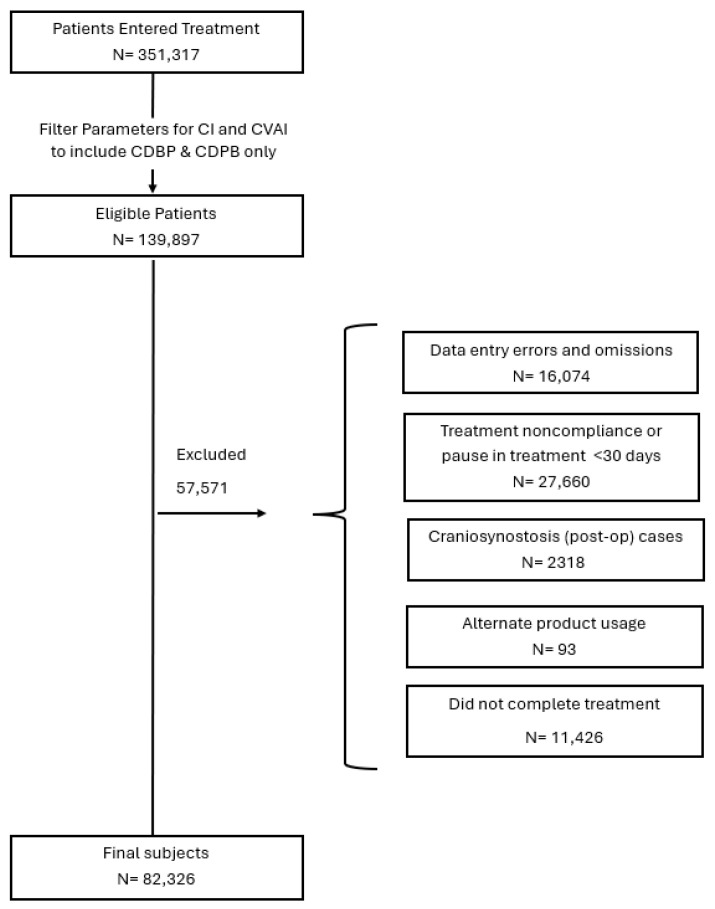
Flow Chart.

Patients were included in this study if they met the anthropometric parameters for cDPB/cDBP, which included both an initial CVAI(S) greater than 3.5 and an initial CI greater than 90%. Other inclusion criteria were an entry age between 3 and 18 months and overall compliance with the treatment protocol (23 h/day use) as reported by parents/caregivers. Patients were excluded from the study if they had: (a) previous treatment with a different CRO, (b) presented as syndromic or with craniosynostosis, (c) documented non-compliance with the treatment protocol, (d) record duplications, (e) incomplete data records, and (f) data entry or validation errors (Figure 1). Patients were sub-grouped by (a) entry age (3 ≤ 4 months, 4 ≤ 6 months, 6 ≤ 8 months, 8 ≤ 11 months, and >11 months), which are consistent with age groupings used in previous studies and (b) a severity rating based on CVAI(S) and CI measurements.

### 2.4. Classification Scale Development for Combinational Head Shapes

Subjects were categorized into four severity tiers—mild, moderate, severe, or very severe—utilizing a specialized classification scale for cDPB/cDBP developed specifically for this research (Table 1). This framework integrates the CVAI classifications from the Children’s Healthcare of Atlanta scale (CHOA scale) with CI severity thresholds established for this study.

This scale expands upon previous descriptive models for combinational cranial morphologies, which classified patient populations using CVAI(L) and CI [4]. However, the present classification framework introduces a more granular five-tier system for brachycephaly, replacing the traditional four-category model, and incorporates refined CI severity thresholds. Furthermore, in accordance with the methodology established by Pinyot, this framework designates combinational cranial deformities based on the predominant morphological feature—either brachycephaly or plagiocephaly—to facilitate more precise clinical characterization. If the severity category as determined by the CVAI (S) and CI is the same, then the naming convention will be represented by cDPB.

### 2.5. Statistical Analysis

The primary outcome measures in this study were defined as the mean change in CVAI(S) and CI from baseline to treatment conclusion. To evaluate the significance between entry and exit measurements, paired t-tests were performed for the aggregate cohort and further stratified by the following five age-based subgroups: 3 ≤ 4 months, 4 ≤ 6 months, 6 ≤ 8 months, 8 ≤ 11 months, and >11 months).

Multiple linear regression (MLR) models were constructed to identify predictor variables associated with changes in CVAI(S) and CI: (1) initial CVAI(S), (2) initial CI, (3) entry age, (4) sex, (5) torticollis or neck involvement, (6) requirement for orthotic remake, (7) laterality of plagiocephaly, (8) prematurity, and (9) multiple birth status.

All statistical tests were two-tailed, and the threshold for statistical significance was established at α = 0.05. Data processing and statistical analyses were conducted using R 4.3.1 (The R Foundation for Statistical Computing, Vienna, Austria) and Microsoft Excel (Microsoft Corp., Redmond, WA, USA).

## 3. Results

### 3.1. Sample Demographics

Of the 351,317 infants who initiated treatment between January 2014 and March 2025, a subset of N = 82,326 infants met the inclusion criteria for combinational deformational plagiocephaly with brachycephaly or combinational deformational brachycephaly with plagiocephaly (cDPB/cDBP) and were included in the final analysis (Figure 1). Consistent with established epidemiological data showing a higher prevalence of cranial deformities in male infants [27,29,30], the cohort was predominantly male (65.8%). The mean age at the start of treatment was 6.3 months, with nearly half of the sample (48.6%) beginning treatment between 4 to <6 months of age. 26.8% of the cohort was born prematurely.

Clinical comorbidities included neck involvement/torticollis (61.1%) as recorded at a patient’s initial consultation visit. Left-sided plagiocephaly was observed in 32.7% of the subjects. The mean treatment duration for the overall cohort was 3.5 months; however, treatment length varied by age at enrollment. Infants who began treatment before 6 months of age required an average duration of approximately 3 months, whereas those in older age groups had longer treatment courses, averaging 4 months (Table 2). Baseline assessment using the Severity Classification Scale for Combinational Plagiocephaly and Brachycephaly showed that moderate cDBP (24.4%) and moderate cDPB (19.4%) were the most common clinical presentations (Table 3).

### 3.2. Change in CVAI(S) and CI

The primary outcome measures utilized to quantify improvement in cranial asymmetry and disproportionality were the mean changes in pre-treatment and post-treatment scores for CVAI(S) and CI, respectively. The mean reduction in CVAI(S) across all age cohorts was −3.64 (95% CI [−3.63, −3.65]; *p* < 0.001). Stratification by age group demonstrated the following reductions: 3–4 months (−5.274, 95% CI [–5.36, −5.20]), 4–6 months (−4.074; 95% CI [−4.09, −4.05]), 6−8 months (−3.262; 95% CI [−3.28, −3.24]), 8−11 months (−2.692; 95% CI [−2.72, 02.66]), and >11 months (−2.247; 95% CI [−2.31, −2.19]); all findings were statistically significant at *p* < 0.001 (Table 4).

Analysis of variance (ANOVA) indicated significant differences in CVAI(S) reduction between age groups (F = 2432.249, *p* < 0.001). Subsequent post hoc analysis with Bonferroni corrections confirmed that all pairwise comparisons between age cohorts reached statistical significance (*p* < 0.001). For the Cephalic Index, the mean reduction was −3.84 (95% CI [−3.83, −3.86]; *p* < 0.001) for the aggregate cohort, with improvements found to be independent of age (Table 4).

**Figure 2:**
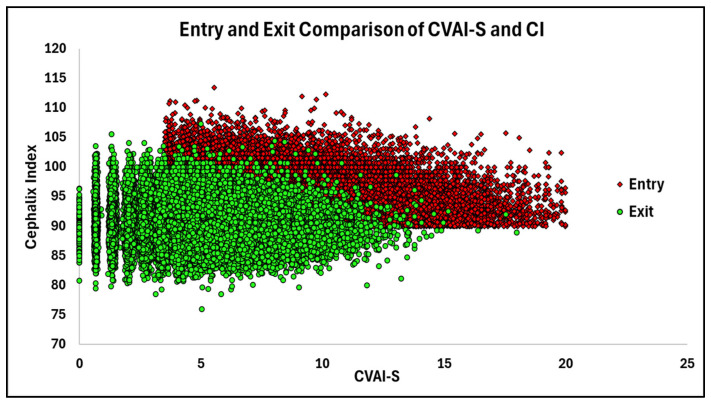
Scatterplot of CVAI(S) and CI at Entry versus Exit.

Scatterplot analysis of pre- and post-treatment measurements for CI and CVAI(S) demonstrated that a majority of the cohort moved toward values within normal limits by the end of treatment (Figure 2).

**Figure 3:**
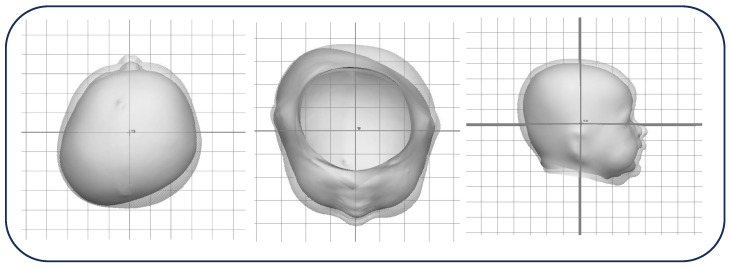
Vertex, subvertex, and lateral view of a 3.75-month-old before and after 1.75 months of treatment with entry measurements of (100.8)-(19.2)-Right and exit measurements of (94.4)-(5.7)-Right.

Figure 3 illustrates the clinical progression of an infant with very severe cDPB who initiated cranial remolding orthosis (CRO) therapy at 3.75 months and received treatment for a total of 1.75 months. The morphological expression of the deformity is characterized by the framework (CI)-(CVAI(S))-Laterality, where this patient had an initial presentation of (100.8) – (19.2) – Right improved to (94.4) – (5.7) – Right at the conclusion of therapy.

### 3.3. Clinical Predictive Factors Associated with Change in CVAI(S)

A multiple linear regression (MLR) analysis was performed to identify clinical predictors associated with change in CVAI(S) scores (Table 5). The resulting model was highly significant F(6, 82,319) = 18,801.21 (*p* < 0.001), with an adjusted R^2^ of 0.58. Several factors were identified as significant independent predictors of the change in CVAI(S) (*p* < 0.001): entry age (β = 0.008), left-sided plagiocephaly (β = −0.420), initial cephalic index (β = −0.056), initial CVAI(S) (β = −0.479), prematurity (β = −0.189), and the presence of torticollis/neck muscle involvement (β = 0.053). Babies with torticollis/neck muscle had a less favorable outcome in terms of change in CVAI(S), while younger age, left-sided plagiocephaly, prematurity, and higher initial severity rating of CI and CVAI(S) were associated with greater reduction in CVAI(S).

### 3.4. Clinical Predictive Factors Associated with Change in CI

An additional multiple linear regression (MLR) analysis was performed to identify clinical predictors associated with the change in CI (Table 6). The resulting model was statistically significant, F(7, 82,318) = 3263.49 (*p* < 0.001), with an adjusted R^2^ of 0.22. The following factors were identified as significant independent predictors (*p* < 0.001) of CI reduction: initial CI (β = −0.335), left-sided plagiocephaly (β = −0.332), multiple birth (β = −0.290), male sex (β = −0.158), initial CVAI(S) (β = −0.049), premature (β = −0.086), and the presence of neck muscle involvement (β = −0.106).

Although entry age reached statistical significance in the preliminary analysis, it was excluded from the final regression model, as supplemental analyses demonstrated a lack of correlational directionality across group comparisons and its very small Beta coefficient. The removal of this variable yielded no appreciable impact on the model’s adjusted R^2^ value, and shifts in the beta coefficients of the remaining variables were negligible (Δ < 0.01).

### 3.5. Clinician-Reported Rating of Outcome

To evaluate treatment efficacy, clinicians utilized a four-point Likert scale with the ratings “poor,” “fair,” “good,” and “great.” These qualitative ratings complement quantitative measurements, as standard linear anthropometric data may not fully capture the complex, three-dimensional changes in head shape.

Across the cohort of infants who began treatment for cDBP or cDPB between 3 and 18 months of age, clinician-rated outcomes of “good” or “great” ranged from 12.9% to 100%, depending on age group and severity, with the highest success of 95–100% of babies in the 3–6 month age group making a “good” or “great” outcome. For infants aged 6–11 months, 82% to 100% received a “good” or “great” rating. Overall, infants with cDPB had higher rates of positive outcomes than those with cDBP. Notably, all infants aged 4–11 months with severe or very severe cDPB were rated “good” or “great.” Infants with cDBP also showed high success, with over 91% rated “good” or “great,” but a clear age-related decline was observed. For infants who started treatment after 11 months of age, fewer than 19% achieved a “good” or “great” outcome (Table 7).

### 3.6. Pre- and Post-Treatment Severity Classification

Post-treatment outcomes highlight the complexity of managing different severity levels of combinational deformities. All patients in the cohort entered treatment within one of the initial six-tier combinational severity classification categories. By the end of treatment, patients’ final head shape was classified into one of sixteen distinct clinical categories, which include isolated plagiocephaly and isolated brachycephaly (Table 8). Correction of asymmetry was very consistent in patients with plagiocephaly-dominant deformities, with 96.0% improving their CVAI by at least one category. In contrast, similar improvements in head proportion were less common (76.7%) among patients with brachycephalic-dominant deformities, and these improvements were closely linked to the initial CI severity (Table 8).

## 4. Discussion

This large retrospective study found that cranial remolding orthosis (CRO) treatment is effective for infants with combined cranial deformities (cDPB/cDBP), resulting in significant improvements in asymmetry (CVAI(S)) and disproportionality (CI) across all age groups. This study represents the largest cohort to date evaluating treatment outcomes and predictive factors specifically in infants with combined head shape deformities, addressing a notable gap in the existing literature.

Consistent with prior studies involving cases of isolated plagiocephaly and isolated brachycephaly, younger age at treatment initiation was associated with greater improvements in cranial asymmetry and shorter treatment durations [3,18,19,20,21,25,28,31,32,33,34,35,36]. In this study, we found that infants with cDPB/cDBP who began treatment earlier demonstrated larger reductions in CVAI(S), reinforcing the importance of early intervention, ideally with a referral at 3–4 months of age, during the early periods of rapid cranial growth. In contrast, we found that age at initiation was not meaningfully associated with change in CI. This suggests that while correction in asymmetry is highly age-sensitive, improvements in cranial disproportionality in cDPB/cDBP may be less dependent on age and more strongly influenced by initial head shape severity, morphology, and the babies’ ability to roll over independently which typically occurs at 4–5 months of age and coincides with reduce time spent in the supine position. Further research regarding the predictive factors that influence correction in disproportionality is recommended, as our regression model for change in CI had an R^2^ value of 0.22, indicating that the model was able to explain only a minimal amount of variation in the change in CI.

The estimate for CVAI(S) reduction in this study of −3.64 ± 0.01 is very similar to Trebilcock et al. estimate of CVAI(S) reduction of −3.42 ± 0.01 in their isolated plagiocephaly study of 27,990 infants [27]. The narrow confidence intervals around the reduction in CVAI(S) parameter estimate indicate that there is a high degree of precision in these estimates. Our estimate for reduction in CI of −3.84 ± 0.01 is slightly less than Kelly et al. estimate of CI reduction of −5.6 ± 2.3 in their isolated brachycephaly study of 4205 infants. This finding shows that there are differences in the growth adjustment approach for correcting cDPB/cDBP as compared with isolated brachycephaly. The strategy for the adjustment pattern for cDPB/cDBP prioritizes a contralateral adjustment for correction of asymmetry while concurrently addressing disproportionality. Once full asymmetry correction is achieved, then the adjustment strategy can be changed to a central occipital adjustment approach for disproportionality correction only. Additionally, it is hypothesized that a disproportionately wide head shape (brachycephaly) requires more volume expansion than asymmetry to correct the head shape, requiring more comprehensive head growth and, often, longer treatment periods, which may also explain these findings.

Multiple linear regression analyses identified several factors that significantly influenced treatment outcomes. Logically, greater initial severity (higher CVAI(S) and CI values) was associated with larger absolute improvements, demonstrating the ability of CRO treatment to produce meaningful correction even in severe cases. Prematurity and neck involvement were also significant predictors, underscoring the multifactorial nature of cranial deformities and the importance of considering comorbid conditions in treatment planning. Meanwhile, the finding that left-sided plagiocephaly was associated with a greater reduction in CVAI(S) and CI measurements is consistent with the CVAI(S) correction finding from an isolated plagiocephaly study [27]. The explanation for the improved left-sided plagiocephaly correction phenomenon requires more examination, as left-sided plagiocephaly may be related to a variety of factors such as greater initial severity or neck muscle involvement. The results from these regression analyses could also be used in future research to develop predictive head shape image modeling and predictive equations, based upon a baby’s initial clinical presentation.

Clinician-reported outcome ratings complemented the anthropometric measures and provided additional insight into overall head shape improvement. A high proportion of infants treated before 8 months of age were rated as having “good” or “great” outcomes, regardless of initial severity, and success rates declined substantially among infants initiating treatment after 11 months. These findings emphasize the importance of early referral and intervention, particularly for infants with combinational deformities. Clinician-rated outcomes are particularly beneficial in infants with cDPB/cDBP because these head shapes present complex three-dimensional growth patterns that are not fully captured by anthropometric measurements alone. A recommendation for future research would be to examine the use of 3D volume parameters to quantify improvements in cDPB/cDBP [37], as 3D stereophotogrammetrics may provide more insight than 2D linear anthropometrics.

A secondary objective of this study was to propose a standardized severity classification system for combinational head shapes. By integrating established CVAI(S) thresholds with CI severity thresholds developed for this study, the proposed classification framework provides a clinically meaningful and reproducible method for characterizing cDPB/cDBP severity. This approach builds upon prior studies’ efforts to categorize mixed head shapes and offers a foundation for more consistent classification, outcome prediction, and comparison across studies [4,8]. However, it is important to note that the classification scale requires further validation among different populations and ethnicities as standards for brachycephaly may differ.

Several limitations should be acknowledged. The retrospective design and reliance on data from a single CRO provider may limit generalizability. Notably, the absence of a control group/no CRO intervention group limits the ability to compare outcomes against the natural course of untreated head shape deformities. The retrospective nature of this study made it unfeasible to include a control group. Furthermore, treatment durations were most likely overestimated due to differences in how treatment dates were recorded, specifically with respect to the point at which the treatment exit occurred. Another limitation is that the data capture method for the presence of torticollis/neck muscle involvement as diagnosed by a physician was only recorded at the initial consultation, potentially leading to an underestimation of the true prevalence of torticollis in this study. Often, torticollis/neck muscle involvement is first recognized by the CRO clinician at the initial consultation and patients are subsequently referred to their pediatricians for physical/occupational therapy intervention. We also did not explore neurodevelopment disorders as a risk factor, as we do not maintain longitudinal records of neurodevelopment status for patients. Finally, the patient data were analyzed without correcting for prematurity, which may limit the comparability of our findings to other studies.

## 5. Conclusions

This study proposes new terminology and a new classification method for infants with combinational head shape deformities for cDPB and cDBP. This classification system streamlines the diagnostic classifications to describe these head shapes. Findings indicate that CRO treatment is effective for infants with cDPB/cDBP, with earlier treatment initiation associated with greater asymmetry correction, shorter treatment durations, and positive clinician-rated outcomes. These findings: (a) support early identification and referral for CRO treatment for babies with combinational head shape deformities, (b) demonstrate the efficacy of CRO treatment for this population, (c) reinforce the value of holistic clinician assessment alongside anthropometric metrics, and (d) contribute a large-scale evidence base to inform proactive clinical decision-making for effectively treating this highly prevalent, yet understudied, head shape deformity in babies.

## Figures and Tables

**Table 1:** Severity Classification Scale for Combinational Plagiocephaly and Brachycephaly.

		Cephalic Index				
		Normal	Mild	Moderate	Severe	Very Severe
		<88%	88–90	90–95	95–100	>100
CVAI(S)	Mild	Mild P	Mild cDPB	Mod cDBP	Sev cDBP	VS cDBP
	≥3.5 to ≤6.25					
	Moderate (Mod)	Mod P	Mod cDPB	Mod cDPB	Sev cDBP	VS cDBP
	>6.25 to ≤8.75					
	Severe (Sev)	Sev P	Sev cDPB	Sev cDPB	Sev cDPB	VS cDBP
	>8.75 to ≤11.0					
	Very Severe (VS)	VS P	VS P	VS cDPB	VS cDPB	VS cDPB
	>11.0					

**Table 2:** cDPB/cDBP Patient Demographics and Clinical Characteristics Categorized Overall and by Entry Age.

	All Ages	3 ≤ 4 Months	4 ≤ 6 Months	6 ≤ 8 Months	8 ≤ 11 Months	>11 Months
N	82,326	3454	40,133	25,853	10,673	2213
Consult age	155.253	-	-	-	-	-
Entry age	191.167	-	-	-	-	-
Consult to Entry Lapse	35.914	28.073	29.089	38.374	50.903	70.890
Treatment Duration (days)	105.771	93.160	95.836	114.200	122.930	124.388
Left Plagiocephaly	26,955	1094	13,091	8565	3450	755
Male	54,188	2344	26,845	16,737	6817	1445
Female	28,138	1110	13,288	9116	3856	768
Torticollis	13,911	821	7288	4058	1478	266
Neck Involvement	36,383	2106	19,367	10,478	3763	669
Multiple Birth	7085	203	3304	2394	967	217
Multiple Bands	17,431	1315	9798	4598	1538	182
Hematoma	881	74	484	217	94	12
Premature	22,094	549	10,130	7382	3296	737
Initial CVAI(S)	7.510	8.912	7.768	7.205	6.913	7.079
Final CVAI(S)	3.869	3.634	3.695	3.944	4.220	4.831
Mean Change in CVAI ***	−3.641	−5.278	−4.074	−3.262	−2.692	−2.247
Initial CVA	−2.303	11.155	10.122	9.685	9.522	9.981
Final CVA	7.643	8.021	7.595	7.586	7.678	8.446
Mean Change in CVA	−2.303	−3.134	−2.527	−2.099	−1.844	−1.535
Initial CI	94.235	94.050	94.298	94.260	94.038	94.061
Final CI	90.394	90.648	90.429	90.277	90.362	90.868
Change in CI ***	−3.842	−3.402	−3.869	−3.983	−3.676	−3.193
Consult Circum (mm)	428.121	406.324	421.187	433.277	443.224	454.834
Exit Circum	454.150	440.401	448.742	458.217	465.273	472.518
Change in Circumference	26.028	34.077	27.555	24.940	22.049	17.683

*** indicates *p* < 0.001.

**Table 3:** Pre-treatment Severity Classification.

	Normal CI < 88	Mild 88 ≤ CI < 90	Moderate 90 ≤ CI < 95	Severe 95 ≤ CI < 100	Very Severe CI ≥ 100

Normal	-	-	-	-	-
CVAI < 3.5					
Mild	-	-	20,128	10,866	2682
3.5 ≤ CVAI ≤ 6.25					
Moderate	-	-	16,026	6451	1446
6.25 < CVAI ≤ 8.75					
Severe	-	-	9945	3494	735
8.75 < CVAI ≤ 11.0					
Very Severe	-	-	7674	2415	464
CVAI > 11.0					

**Table 4:** Mean change in CVAI and CI by Age at Initiation of Treatment.

	All	3 ≤ 4	4 ≤ 6	6 ≤ 8	8 ≤ 11	>11
Change in CVAI ***	−3.641	−5.278	−4.074	−3.262	−2.692	−2.247
Change in CI ***	−3.842	−3.402	−3.869	−3.983	−3.676	−3.193

*** denotes statistical significance at *p* < 0.001.

**Table 5:** CVAI(S): Multiple Linear Regression Analysis.

Regression Statistics						
Multiple R	0.760					
R Square	0.578					
Adjusted R Square	0.578					
Standard Error	1.292					
Observations	82,326					
ANOVA						
	df	SS	MS		F	Significance F
Regression	6	188,173.187	31,362.198		18,801.199	0.000
Residual	82,319	137,315.965	1.668			
Total	82,325	325,489.152				
	Coefficients	Standard Error	t Stat	p-value	Lower 95%	Upper 95%
Intercept	3.761	0.141	26.665	0.000	3.484	4.037
Entry Age	0.008	0.000	102.013	0.000	0.008	0.008
Left Side	−0.420	0.010	−43.612	0.000	−0.439	−0.402
Initial CI	−0.056	0.001	−38.165	0.000	−0.058	−0.053
Initial CVAI(S)	−0.479	0.002	−293.883	0.000	−0.482	−0.476
Premature	−0.189	0.010	−18.478	0.000	−0.209	−0.169
Neck Involvement	0.053	0.009	5.583	0.000	0.034	0.071

**Table 6:** CI—Multiple Linear Regression Analysis.

Regression Statistics						
Multiple R	0.466					
R Square	0.217					
Adjusted R Square	0.217					
Standard Error	2.011					
Observations	82,326					
ANOVA						
	df	SS	MS		F	Significance F
Regression	7	92,348.982	13,192.712		3263.489	0
Residual	82,318	332,771.944	4.043			
Total	82,325	425,120.927				
	Coefficients	Standard Error	t Stat	p-value	Lower 95%	Upper 95%
Intercept	28.398	0.217	131.027	0.000	27.974	28.823
Male	−0.158	0.015	−10.710	0.000	−0.187	−0.129
Left Side	−0.332	0.015	−22.118	0.000	−0.362	−0.303
Initial CI	−0.335	0.002	−148.031	0.000	−0.339	−0.331
Initial CVAI(S)	−0.049	0.003	−19.324	0.000	−0.054	−0.044
Premature	−0.086	0.017	−5.077	0.000	−0.119	−0.053
Multiple Birth	−0.290	0.027	−10.807	0.000	−0.343	−0.237
Neck Involvement	−0.106	0.015	−7.236	0.000	−0.135	−0.077

**Table 7:** Clinician-reported Rating of Good or Great Outcome at Exit based on Age and Initial Severity.

		3 ≤ 4		4 ≤ 6		6 ≤ 8		8 ≤ 11		>11	
		Months		Months		Months		Months		Months	
cDBP	Moderate	435	97.5%	8449	96.6%	6642	94.6%	2863	88.0%	505	15.5%
	Severe	509	96.6%	7957	96.4%	5319	93.3%	2046	85.1%	309	12.9%
	Very Severe	168	95.5%	2301	95.2%	1431	91.1%	455	82.4%	101	18.3%
cDPB	Moderate	656	97.3%	7406	96.5%	4637	92.9%	1866	83.9%	315	14.2%
	Severe	744	97.1%	6833	100.0%	3500	100.0%	1127	100.0%	185	16.4%
	Very Severe	842	97.3%	5561	100.0%	2411	100.0%	621	100.0%	96	15.5%

**Table 8:** Pre and Post Treatment Severity Classifications.

Pre-Treatment Severity						Post-Treatment Classification	
cDBP			cDPB				
Moderate	Severe	Very Severe	Moderate	Severe	Very Severe		
1630	417	23	3602	2361	1098	Mild	cDPB
11	182	33	642	2251	2720	Moderate	
0	0	4	4	122	938	Severe	
1	0	0	0	2	179	Very Severe	
1662	3819	538	3312	3331	1580	Moderate	cDBP
11	703	840	39	312	376	Severe	
0	2	83	2	0	21	Very Severe	
925	96	5	2531	1926	1204	Mild	Isolated Plagio
4	4	0	162	782	1300	Moderate	
2	0	0	0	24	401	Severe	
0	0	0	0	0	72	Very Severe	
6024	1357	108	2037	620	167	Mild	Isolated Brachy
5766	9097	1491	2036	1071	296	Moderate	
49	1298	1577	32	106	47	Severe	
0	3	139	1	0	1	Very Severe	
4043	339	22	1626	531	153	Within Normal Limits	
20,128	17,317	4863	16,026	13,439	10,553	Total Patients (N)	

## Data Availability

The data analyzed in this study is part of Cranial Technologies’ electronic health record (EHR) and is protected by privacy restrictions. The data cannot be made publicly available.

## Abbreviations

The following abbreviations are used in this manuscript:

CRO	Cranial remolding orthosis
cDPB	Combinational deformational plagiocephaly with brachycephaly
cDBP	Combinational deformational brachycephaly with plagiocephaly
CVAI	Cranial vault asymmetry index
CVA	Cranial vault asymmetry
